# DDX3X: structure, physiologic functions and cancer

**DOI:** 10.1186/s12943-021-01325-7

**Published:** 2021-02-24

**Authors:** Jie Mo, Huifang Liang, Chen Su, Pengcheng Li, Jin Chen, Bixiang Zhang

**Affiliations:** 1 Hubei Key Laboratory of Hepato-Pancreato-Biliary Diseases, Wuhan, Hubei 430030 People’s Republic of China; 2 Hepatic Surgery Centre, Tongji Hospital, Tongji Medical College, Huazhong University of Science and Technology, Wuhan, Hubei 430030 People’s Republic of China; 3 Clinical Medicine Research Centre for Hepatic Surgery of Hubei Province, Wuhan, Hubei 430030 People’s Republic of China; 4 Key Laboratory of Organ Transplantation, Ministry of Education, P.R.China; Key Laboratory of Organ Transplantation, National Health Commission, P.R.China; Key Laboratory of Organ Transplantation, Chinese Academy of Medical Sciences, Wuhan, China

**Keywords:** DDX3X, RNA metabolism, Cancer

## Abstract

The DEAD-box helicase family member DDX3X (DBX, DDX3) functions in nearly all stages of RNA metabolism and participates in the progression of many diseases, including virus infection, inflammation, intellectual disabilities and cancer. Over two decades, many studies have gradually unveiled the role of DDX3X in tumorigenesis and tumour progression. In fact, DDX3X possesses numerous functions in cancer biology and is closely related to many well-known molecules. In this review, we describe the function of DDX3X in RNA metabolism, cellular stress response, innate immune response, metabolic stress response in pancreatic β cells and embryo development. Then, we focused on the role of DDX3X in cancer biology and systematically demonstrated its functions in various aspects of tumorigenesis and development. To provide a more intuitive understanding of the role of DDX3X in cancer, we summarized its functions and specific mechanisms in various types of cancer and presented its involvement in cancer-related signalling pathways.

## Background

The DEAD (Asp-Glu-Ala-Asp)-box helicase family is the largest helicase family in eukaryotes and functions in nearly all aspects of eukaryotic RNA metabolism [1, 2]. DDX3 is one of the members of the DEAD-box helicase family. The human genome encodes two types of *DDX3* genes: *DDX3X* and its homologue *DDX3Y* [3]. *DDX3X (DBX, DDX3)* is located on p11.3–11.23 on the X chromosome and escapes X-inactivation in females [4, 5]. It is expressed ubiquitously in human tissues and participates in many biological processes [2, 6–8]. *DDX3Y (DBY)* is located on the Y-chromosomal AZFa region [9]. Unlike its multifunctional homologue, it is only expressed in spermatocytes by translation control and is crucial for spermatogenesis [9, 10]. The two proteins share 92% similarity in protein sequence identity. Although their range of expression and function seems quite different, several lines of evidence indicate that DDX3X and DDX3Y might be interchangeable in some circumstances [11, 12]. In this review, we mainly discuss the structure, localization and functions of DDX3X. The DDX3 subfamily of DEAD-box helicases includes human DDX3X, yeast Ded1p, Xenopus An3, mouse PL10 and *Drosophila* Belle. The structures of these homologous proteins are highly conserved, indicating their crucial role in biological processes in life [13]. As an outstanding member of the DEAD-box family, DDX3X is able to regulate nearly all stages of RNA metabolism, including transcription, pre-mRNA splicing, RNA export and translation [14–20]. Based on its function in RNA metabolism, DDX3X has a major effect on many biological processes. Dysfunction of this helicase plays a vital role in various diseases, including viral infection, inflammation, intellectual disability and cancer [7, 8, 21–23].

Over the years, DDX3X has become a molecule of interest in cancer biology. Many studies in over 10 types of cancers gradually uncovered its functions in the progression of malignancies [23–32]. In fact, DDX3X has a wide range of functions, ranging from tumorigenesis to metastasis [24, 33–36]. In these processes, DDX3X is also closely associated with many other well-known molecules in cancer-related pathways, including P53, β-catenin and KRAS [25, 26]. However, whether it functions as an oncogene or a tumour suppressor has always been controversial. In this review, we first describe its functions in RNA metabolism and other biological processes. Then, we focus on the role of DDX3X in cancer biology and systematically demonstrate its functions in various aspects of tumorigenesis and development. To provide a more comprehensive understanding of its role in cancer, we summarized the role of DDX3X and the specific mechanisms in various types of cancer and presented its involvement in cancer-related signalling pathways.

## Overview of DDX3X

### Structure of DDX3X

**Fig. 1:**
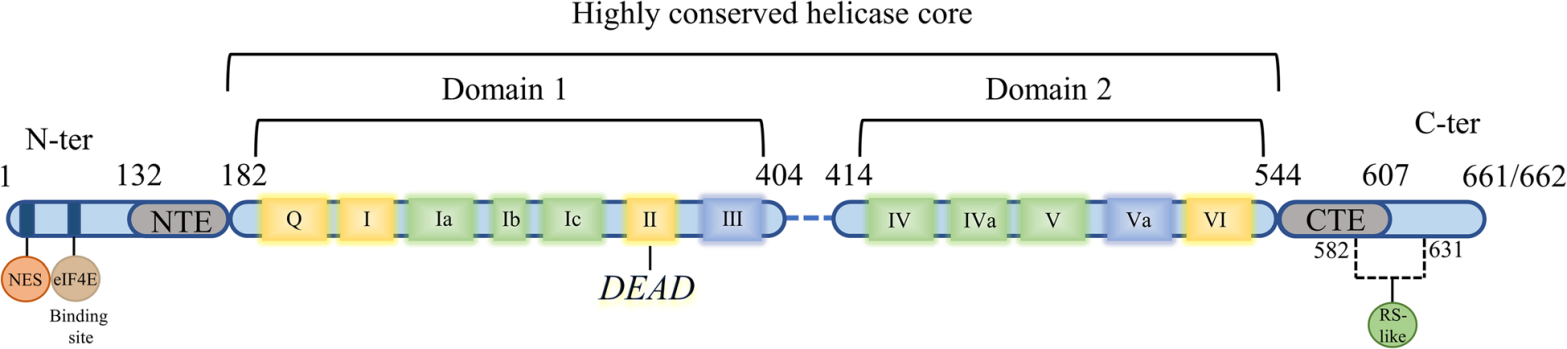
The structure of DDX3X. DDX3X can be divided into three regions: the highly conserved helicase core, the N-terminus and the C-terminus. The helicase core includes 12 signature helicase motifs that are involved in ATP binding and hydrolysis (yellow), RNA binding (green) and communication between RNA and ATP binding sites (bule). The helicase core along with the NTE and CTE are the minimal functional core of the Ded1/DDX3 subfamily. The NES residing in the N-terminus is involved in CRM-1-mediated nuclear export of DDX3X. The eIF4E binding site is located near amino acid 40. The RS-like (arginine/serine-like) region residing in the C-terminus is responsible for interacting with the nuclear export receptor TAP

The DDX3X protein is 662- or 661-amino acid polypeptide (55 kD) depending on alternative splicing [6]. As a member of the DEAD-box helicase family, DDX3X contains a highly conserved helicase core that is shared with all other DEAD-box helicases [10]. The helicase core is comprised of two RecA-like domains named domain 1 and domain 2. These two domains contain 12 signature helicase motifs that are involved in ATP binding and hydrolysis (motifs Q, I, II/DEAD, VI), RNA binding (Ia, Ib, Ic, IV, IVa, V, VI) and communication between RNA and ATP binding sites (III, IVa) [10, 37] (Fig. 1). In cancer biology, RNA helicase activity has been confirmed to be crucial for the promotion of cyclin E1 and Rac1 translation, whereas ATPase activity is important for P21 transcription [20, 33, 36]. The helicase core is flanked by the N-terminus and C-terminus, the sequences of which distinguish the helicases from another. Unlike the restricted highly conserved helicase core, both the N- and C-termini contain several characteristic sequence regions. In DDX3X, an nuclear export signal (NES) resides in the N-terminus, which is involved in CRM-1-mediated nuclear export of DDX3X [38, 39] (Fig. 1), whereas the C-terminus contains an RS-like (arginine/serine-like) region that is responsible for interacting with the nuclear export receptor TAP [16] (Fig. 1). In addition, a putative eIF4E binding site, which is absent in other DEAD-box helicases, resides around amino acid 40 [18] (Fig. 1). In a recent study, the minimal functional core of the Ded1/DDX3 subfamily (including DDX3X, Ded1p, and Vasa/DDX4) was redefined to contain not only two RecA-like domains but also two short extensions named the N-terminal extension (NTE) and the C-terminal extension (CTE), which flank the RecA-like domains [40] (Fig. 1). The NTE is involved in ATP hydrolysis, while the CTE is essential for RNA duplex unwinding [40, 41]. Mutations in natural killer/T-cell lymphoma (NKTCL) and medulloblastoma (MB) mostly cluster in the two RecA-like domains [32, 42]. In T-cell acute lymphoblastic leukaemia (T-ALL), the fusion of *DDX3X* with *MLLT10* preserves the N-terminus of DDX3X, which contains the NES and eIF4E binding site [31]. As a multifunctional protein, DDX3X associates with many other molecules to perform different functions (Table 1).

**Table 1:** DDX3X and its binding proteins

Interaction protein	Interaction region	Function	Ref
ALKBH5	211–404	Modulating m^6^A RNA Demethylation	[43]
CK1ε	456–662	Involving in WNT/β-catenin signaling	[44]
CRM1	260–517	Promoting Rev./REE-CRM1-dependent export of HIV transcripts	[39]
eIF4E	38–44	Involving in translation initiation	[18]
GSK3-β	100–662	Forming anti-apoptosis complex	[45]
PABP1	227–534	Involving in stress granules assembly	[46]
TAP/NXF1	536–662	Transported by TAP with mRNPs	[16]

### Cellular localization of DDX3X

Although DDX3X is present in both the cytoplasm and nucleus, most studies indicate that it predominantly localizes to the cytoplasm [16, 17, 39, 47]. In fact, the helicase possesses nucleocytoplasmic shuttling properties via its association with CRM1 and TAP, and its nuclear localization is closely related to the efficiency of CRM1-mediated export [16, 39, 48]. Importantly, the change in the cellular localization of DDX3X in normal tissues might lead to tumorigenesis [20, 49]. Concrete subcellular localization of DDX3X to organelles, including the nucleolus, centrosome and mitochondria, has also been demonstrated; it performs different functions based on its location [4, 48, 50]. Additionally, DDX3X is also present in intracellular RNA/protein bodies such as stress granules [46].

## Biological functions of DDX3X

### RNA metabolism

#### Transcription

DDX3X enhances transcription by interacting with transcription factors to promote their binding to the promoter of the target gene [20, 25, 26, 51]. The best characterized mechanism is its cooperation with the transcription factor SP1. The downstream genes of DDX3X-SP1-mediated transactivation include P21, KRAS, and MDM2 [20, 25, 26], which are critical for cancer development and progression. DDX3X also interacts with YY1 to facilitate the transcription of genes involved in WNT/β-catenin signalling [51]. Moreover, DDX3X can directly impinge on E-cadherin and IFN-β promoters to regulate their transcription without cooperating with any transcription factor [24, 52].

#### Pre-mRNA splicing

DDX3X has been successively identified in affinity-purified human spliceosomes, messenger ribonucleoproteins (mRNPs) and spliceosomal B complexes [14, 53, 54]. MERZ et al. found that the link between DDX3X and mRNPs is achieved by DDX3X binding with exon junction complex (EJC) proteins [14]. However, the specific function of DDX3X in pre-mRNA splicing needs to be further elucidated.

#### RNA export

DDX3X is involved in facilitating Rev./REE-CRM1-dependent export of HIV transcripts [39]. In this process, DDX3X binds with CRM1 and localizes to nuclear membrane pores [39]. The NES residing in the N-terminus of the helicase is thought to be responsible for binding DEAD-box helicases with CRM1 as a cargo [55]. However, the region responsible for the association between DDX3X and CRM1 is C-terminal residues 260–517 [39]. In addition, the interaction of DDX3X and CRM1 does not require RAN-GTP [39]. Therefore, DDX3X is a functional element in the complex rather than a passenger. DDX3X also interacts with cap-binding protein complex (CBC) and Tip-associated protein (TAP) [16, 56], which are major receptors for bulk mRNA export [57, 58]. Nonetheless, the helicase has little effect on general mRNA export [16]. TAP is recruited to mRNPs and is responsible for their export [58]. Considering the roles of DDX3X and TAP in pre-mRNA splicing, there is a possibility that DDX3X is recruited to mRNPs during splicing, accompanies mRNPs to be exported by TAP and completes its function in RNA metabolism in the cytoplasm. In addition to interacting with CRM1 and TAP, DDX3X also participates in eIF4E-mediated mRNA export [56]. However, its actual role in the process needs to be further explored.

#### Translation

**Fig. 2:**
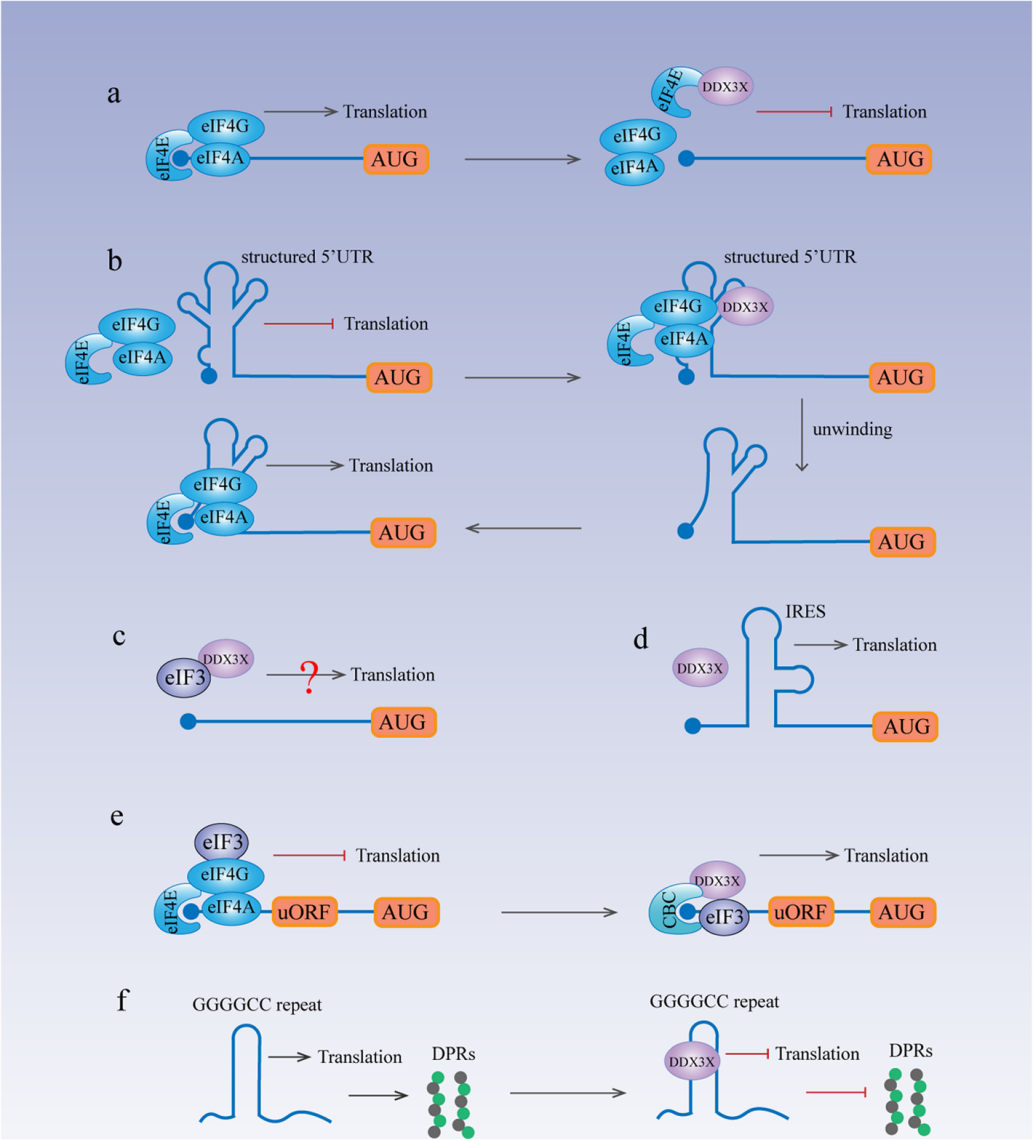
DDX3X and Translation. **a** DDX3X suppresses inhibition by directly binding with eIF4E to inhibit the eIF4E-eIF4G interaction. **b** DDX3X facilitates cap-dependent translation initiation of some RNAs that contain structured 5′UTRs by binding with the eIF4F complex. **c** DDX3X may facilitate translation by interacting with eIF3. **d** DDX3X facilitates IRES-mediated translation of viral RNAs and some cellular transcripts, the specific mechanism of which remains unclear. **e** DDX3X interacts with the cap-binding protein complex (CBC) and eIF3 to promote the translation of uORF-containing mRNAs. **f** DDX3X directly binds to (GGGGCC) n RNAs to suppress RAN translation

Eukaryotes possess two translation initiation mechanisms: cap-dependent and cap-independent translation. Cap-dependent translation starts via recognition of the m^7^GTP cap and the subsequent recruitment of the 43S preinitiation complex (PIC) to the mRNA [59]. This process is facilitated by the eIF4F complex, which consists of eIF4E, eIF4G and eIF4A [59]. While the translation of most cellular mRNAs depends on this process [59], some RNA viruses along with several cellular transcripts utilize cap-independent translation, which requires an internal ribosomal entry site (IRES) on the RNA molecule [59]. DDX3X is involved in both cap-dependent and cap-independent translation initiation to regulate protein synthesis. In liver cancer, DDX3X inhibits the eIF4E-eIF4G interaction by binding with eIF4E to repress global protein synthesis (Fig. 2a). In contrast, it also facilitates cap-dependent translation initiation of some specific RNAs that contain structured 5′UTRs by binding with the eIF4F complex [19, 33, 36] (Fig. 2b). In HeLa cells, DDX3X may facilitate protein synthesis by interacting with eIF3, but the specific role of DDX3X-eIF3 binding in protein synthesis remains unclear (Fig. 2c). In addition to its involvement in cap-dependent translation initiation, DDX3X facilitates IRES-mediated translation of both viral RNA and some cellular transcripts through its unwinding ability and interaction with the eIF4E complex [4, 16, 18, 19, 30]. Another report also stated that DDX3X cooperates with ribosome protein RPL13 and eIF3 subunits e and j to facilitate viral IRES-mediated translation [60] (Fig. 2d).

DDX3X also participates in specialized translation programs. In eukaryotes, upstream open reading frames (uORFs) lead to defects in translation and nonsense-mediated decay (NMD) of transcripts, thereby limiting the expression of key regulators of the stress response and epithelial-mesenchymal transformation (EMT) [61]. DDX3X facilitates the translation of uORF-containing mRNAs by cooperating with the cap-binding protein complex (CBC) and eIF3 to enhance the metastatic ability of cancers [29] (Fig. 2e). Hexanucleotide GGGGCC repeat expansion in the C9ORF72 gene can cause toxic accumulation of dipeptide repeat (DPR) proteins, which is a common cause of amyotrophic lateral sclerosis (ALS) and frontotemporal dementia (FTD) [62]. DPR proteins are produced through an unconventional translation method called repeat-associated non-AUG (RAN) translation [63]. DDX3X effectively inhibits RAN translation by directly binding to (GGGGCC) n RNAs [64] (Fig. 2f). Therefore, it is a potential therapeutic target for ALS/FTD.

#### MicroRNA expression

DDX3X regulates microRNA (miRNA) levels in a direct and an indirect manner [65, 66]. As an RNA binding protein (RNP), DDX3X binds with the miR-20a locus and regulates its expression level [65]. Depletion of DDX3X leads to reductions in miR-20a pri/pre/mature species [65], implying that it is involved in pri-miRNA production or stability. In liver cancer, DDX3X affects the levels of a subset of tumour-suppressive miRNAs by reducing DNMT3A (DNA methyltransferase 3A) binding and hypermethylation on the promoter regions of these miRNAs [66].

### Cellular stress response

When encountering cellular stresses, the cell faces two choices: survival or death. Under cellular stress, stress granules (SGs), which are large cytoplasmic foci comprising RNPs (ribonucleoproteins), are formed to protect cells from death [67]. On the other hand, cellular stressors can activate inflammasomes, which are multiprotein heteromeric complexes, that direct cells to pyroptosis, a form of programmed cell death [68]. DDX3X plays a pivotal role in the crosstalk of these two processes and determines the fate of these cells [69]. DDX3X participates in the assembly of SGs, but it also has the ability to interact with NLRP3 to activate inflammasomes. The assembly of SGs detains DDX3X, thus repressing the activation of the NLRP3 inflammasome. The competition between SGs and NLRP3 for DDX3X determines the ultimate fate of the cell [69].

### Innate immune response

DDX3X plays an important role in the TANK-binding kinase 1 (TBK1)-dependent innate immune response. DDX3X is a substrate of TBK1 [52]. Phosphorylation of DDX3X by TBK1 leads to DDX3X directly interacting with the IFN-β promoter to activate its transcription [52]. Moreover, DDX3X can influence the NF-κB signalling pathway and affect the production of various inflammatory cytokines, such as IL12 and IFNγ [70]. Loss of DDX3X expression in macrophages leads to deficiency in restricting *L. monocytogenes* growth [70].

### Metabolic stress response in pancreatic β cells

The transcription factor JUND can promote β cell apoptosis by regulating pro-oxidant and proinflammatory genes [71]. During metabolic stress, such as high levels of glucose and free fatty acids, JUND expression is upregulated in pancreatic cells via the MEK/ERK/hnRNPK pathway at the posttranscriptional level [72]. DDX3X binds with hnRNPK and is essential for efficient translation of JUND [72].

### Embryo development

The WNT/β-catenin signalling pathway plays an important role in embryonic development [73]. DDX3X participates in this pathway as a regulatory subunit of CK1ε [44]. Under WNT signalling, DDX3X binds to casein kinase 1 ε (CK1ε) and activates its kinase activity. Activated CK1ε then phosphorylates the scaffold protein dishevelled (Dvl), thereby ensuring the formation of the WNT/β-catenin signalosome [44]. Lack of DDX3X expression in *Xenopus* embryogenesis leads to abnormal embryonic development marked by enlarged heads and eyes, shortened tails, and defective melanocyte and eye pigmentation [44]. In mouse embryos, DDX3X is crucial for both extraembryonic and embryonic development [74]. Deficient expression of DDX3X leads to higher levels of genome damage and cell cycle arrest during embryogenesis [74].

## DDX3X in cancer

DDX3X is closely related to several of the hallmarks of cancer, including evading growth suppressors, resisting cell death, activating invasion and metastasis, promoting gene instability and mutation and deregulating cellular metabolism [4, 25, 45, 50, 75, 76]. Here, we first summarized DDX3X protein expression and clinical characteristics in multiple cancers. Then, we described its function as it relates to the hallmarks of cancer. A summary of its functions and specific mechanisms in various types of cancers is listed in Table 2.

**Table 2:** Oncogenic/tumor-suppressive role of DDX3X in various cancers

Cancer type	Oncogenic/tumor-suppressive	Evidence	Mechanism/pathway	Ref
Glioma	Oncogenic	Protein expression; Positively correlated with Snail	-	[77]
			-	[78]
Medulloblastoma (MB)	Oncogenic	Protein expression	-	[79]
		Mutations	Mutations led to alteration of protein function	[80–82]
		Inhibitor therapy	Inhibiting WNT/β-catenin signaling	[79]
Meningioma	Oncogenic	Protein expression	-	[83]
	Unknown	Mutations	-	[84]
Head and neck squamous cell carcinoma (HNSSC)	Oncogenic	Protein expression	-	[29, 85]
	Tumor-suppressive	Protein expression	-	[49]
		Stemness	-	[86]
		Promoting metastasis	Cooperating with CBC complex and eIF3 to promote ATF4 translation	[29]
		Inhibitor therapy	-	[86, 87]
Cutaneous squamous cell carcinoma (cSCC)	Tumor-suppressive	Protein expression	-	[20]
Melanoma	Oncogenic	Stemness	-	[88]
	Tumor-suppressive	Mutations	Mutations mostly led to loss of function	[30]
		Repressing metastasis	Promoting MITF translation	[30]
Lung cancer	Oncogenic	Protein expression	-	[89]
		Stemness	-	[91]
		Inhibitor therapy	Inhibiting Wnt/β-catenin pathway activity; impairing radiation-induced DNA double-strand break (DSB)	[90]
	Tumor-suppressive	Protein expression	-	[26, 92]
		Repressing proliferation	Synergistically enhancing P53-activated P21 transcription	[92]
		Repressing metastasis	Promoting MDM2 transcription to prevent E-cadherin degradation	[26]
Mesothelioma	Unknown	Mutations	-	[93, 94]
Breast cancer	Oncogenic	Protein expression	-	[95]
		Inducing tumorigenesis	-	[24]
		Hypoxia responsive	Directly regulated by HIF-1α	[96]
		Promoting proliferation	Downregulating KLF4 expression via altering KLF4 mRNA exon usage; downregulating P21	[24, 97]
		Promoting metastasis	upregulating E-cadherin expression via interacting to its promoter	[24]
		Inhibitor therapy	Targeting mitochondria translation	[50]
Hepatocellular carcinoma (HCC)	Oncogenic	Protein expression	-	[90]
		Inducing tumorigenesis	-	[90]
	Tumor-suppressive	Protein expression	-	[20, 23]
		Reducing tumorigenesis	Maintaining genome stability	[12]
		Repressing stemness	Upregulating the expression of a subset of tumor-suppressive miRNAs via reducing DNMT3A activity	[66]
		Repressing global protein synthesis	Interacting with eIF4E and inhibiting its activity	[18]
Gallbladder carcinoma	Oncogenic	Protein expression	-	[28]
Pancreatic ductal adenocarcinoma (PDAC)	Oncogenic	Protein expression	-	[27]
Colorectal carcinoma (CRC)	Oncogenic	Protein expression	-	[98, 99]
		Promoting metastasis	DDX3X/KRAS/ERK/AKT/β-catenin/ZEB1 axis; DDX3X/CK1ε/Dvl2 axis; DDX3X/KRAS/HIF-1α/YAP1/SIX2 axis	[100–102]
		Drug resistance	DDX3X/YAP1/SIX2 axis	[102]
		Inhibitor therapy	Inhibiting WNT/β-catenin signaling; mitochondrial swelling and increased ROS production	[98, 99]
	Tumor-suppressive	Protein exprssion	-	[103]
		Repressing metastasis	DDX3X/Snail/E-cadherin axis	[103]
Prostate cancer	Oncogenic	Protein expression	-	[104]
		Inhibitor therapy	Radiosensitizing prostate cancer cell	[104]
Ewing sarcoma	Oncogenic	Protein expression	-	[105]
		Inhibitor therapy	repressing translation of proteins with conserved biologic functions	[105]
Chronic lymphocytic leukemia (CLL)	Unknown	-	-	[106–108]
T-cell acute lymphoblastic leukemia (T-ALL)	Oncogenic	Fusion with *MLLT10*	-	[31, 109]
Natural killer/T-cell lymphoma (NKTCL)	Tumor-suppressive	Repressing proliferation	-	[42]
		Mutations	Abnormal activated NF-κB and MAPK pathways	[42]
Aggressive natural killer-cell leukemia (ANKL)	Unknown	Mutations	-	[110]
Burkitt lymphoma (BL)	Unknown	Mutations	-	[161]
Burkitt-like lymphoma with 11q aberration (BLL-11q)	Unknown	Mutations	-	[111]
Various cell lines: Hela, Huh7, HCT116	Tumor-suppressive	Repressing proliferation	Promoting P21 transcription via interacting with SP1	[20]
Various cell lines: OVCAR3 ES2, A549 H1437, SUM159, HCC1500, HT1080	Tumor-suppressive	Substrate of CMA	-	[162]
Various cell lines: MDA-MB-231, 1321N1, Jurkat, HeLa	Oncogenic	Anti-apoptosis	Cooperating with GSK3 and cIAP-1 to confront with extrinsic apoptosis signaling	[45]
P53 wide-type cell line: MCF-7, SH-SY5Y	Tumor-suppressive	Promoting DNA damage-induced apoptosis	Stabilizing P53 expression via interacting with it	[112]
P53 non-function or mutation cell line: Hela, MDA-MB-231	Oncogenic	Repressing DNA damage-induced apoptosis	-	[112]
Hela cell line	Tumor-suppressive	Promoting proper chromosome segregation	Interacting with hCAP-H	[35]
Various cell lines: HCT116, U2OS	Tumor-suppressive	Ensuring bipolar mitosis	Colocalizing with P53 in centrosome via upregulating P53 expression and phosphorylating P53 to inactivate and coalesce excess centrosome	[4]
Various cell lines: N2A, Hela	Oncogenic	Promoting metastasis	DDX3X/Rac1/β-catenin axis	[36]
Various cell lines: MKN-45, AGS	Oncogenic	Facilitating β-catenin signaling	Transactivating YY1 in the help of *circ-CTNNB1*	[51]
Hela cell line	Oncogenic	Promoting protein synthesis	Interacting with eIF3	[17]
		Anti-apoptosis	Downregulating P21 expression	[113]
Various cell lines: Hela, H1299, A549 and U2OS	Oncogenic	Promoting G1/S phase transition	Promoting cyclin E1 translation	[33]

### Protein expression and clinical characteristics

Over two decades, many cohort studies in various cancers have investigated the expression level of DDX3X and its connection with the clinical characteristics of tumours. However, the results are contradictory [23, 26, 49, 85, 89, 90], which might be caused by the use of different detection methods, different antibodies or the different cut-offs for positivity [23, 26, 49, 85, 89, 90]. To provide a more succinct description, we have summarized the association of DDX3X expression and the clinical characteristics of various tumours in Table 3.

**Table 3:** Expression of DDX3X in various cancers

Cancer type	mRNA/Protein	High/Low expression	Percentage	Clinical characteristics	remarks	ref
Glioma	mRNA and Protein	High	–	Positively correlated with WHO Grading; associated with poor median survival	–	[77]
Medulloblastoma (MB)	Protein	High	55% (31/56) in pediatric 67% (6/9) in adult	–	Mainly in cytoplasm	[79]
Meningioma	Protein	High	–	Significantly higher in atypical meningiomas than in benign meningiomas	Mainly in cytoplasm	[83]
Head and neck squamous cell carcinoma (HNSSC)	Protein Protein Protein	High High Low	71% (15/21) 51% (217/423) 90% (290/324)	Associated with lymph node metastasis (N value), stage and poor patient survival Associated with shorter median survival (HR = 1.34, 95%CI = 1.00–1.81) in smokers Associated with male gender, smoking, alcohol consumption, betel quid chewing, poor RSF and poor OS; associated with poorer OS in non-smokers	Mainly in cytoplasm Subtype: 206 OSCC and 217 OPSSC; Mainly in cytoplasm Subtype: OSCC; Both in cytoplasm and nucleus	[29] [85] [49]
Cutaneous squamous cell carcinoma (cSCC)	Protein	–	–	–	normal epidermis: mainly in nuclear; cSCC: mainly in cytoplasm	[20]
Lung cancer	Protein mRNA Protein	high Low Low	66% (63/94) 53% (73/138) 53% (76/144)	Associated with a shorter survival time (HR = 2.1, 95% CI; 1.13–3.93); a predictor of OS - Associated with a shorter median period of OS (HR = 1.61, 95%CI = 1.04–2.48) and RSF (HR = 1.78, 95%CI = 1.17–2.69)	Mainly in cytoplasm More common in E6 positive or P53 mutation samples; Associated with P21 and E-cadherin expression	[89] [26] [92]
Breast cancer	Protein	High	35% (127/366)	–	Cytoplasm; associated with hypoxia response	[95]
Hepatocellular carcinoma (HCC)	mRNA Protein mRNA	High Low Low	64% (29/45) 57% (49/86) 58% (26/45)	- More common in males and HBV-positive patients -	- - -	[90] [23] [20]
Gallbladder carcinoma	Protein	High	55% (69/126)	Associated with large tumor size, high TNM stage, lymph node metastasis, poor surgical curability and OS	Mainly in cytoplasm	[28]
Pancreatic ductal adenocarcinoma (PDAC)	Protein	High	52% (55/106)	Associated with poor differentiation, surrounding tissue and lymph node metastasis, advanced TMN stage, shorter survival and motility	Mainly in cytoplasm	[27]
Colorectal cancer (CRC)	Protein Protein mRNA	High High Low	41% (124/303) 53% (28/53) -	- - Prognostic predictive indicator (RNA sequencing, HR = 0.53; RNA microarray analysis, HR = 0.72); associated with poor OS and RFS, distant metastasis	Mainly in cytoplasm - -	[98] [99] [103]
Prostate cancer	Protein	High	–	–	Both in cytoplasm and nuclear; positively associated with P21, androgen receptor (AR), PHD2, PHD3, CA9	[104]
Sarcoma	Protein	High	61% (103/170)	–	Mainly in cytoplasm	[105]

Evidence has shown that DDX3X is overexpressed in glioma, medulloblastoma (MB), meningioma, head and neck squamous cell carcinoma (HNSSC), lung cancer, breast cancer, hepatocellular carcinoma (HCC), gallbladder carcinoma, pancreatic ductal adenocarcinoma (PDAC), colorectal cancer (CRC), prostate cancer and sarcoma [27–29, 77, 79, 83, 85, 89, 90, 95, 98, 99, 104, 105]. Among them, lung cancer, gallbladder carcinoma and the smoking subpopulation of patients with HNSSC shows a correlation between overexpression of DDX3X and poor prognosis (overall survival (OS)/relapse-free survival (RFS)/median survival time) [28, 29, 85, 89]. From a pathological point of view, overexpression of DDX3X is positively correlated with pathological classification in glioma, meningioma and PDAC [27, 77, 83], indicating that DDX3X has the potential to differentiate the degrees of pathological classification of tumours. Conversely, a reduction in DDX3X has been reported in HNSSC, lung cancer, HCC, and CRC [20, 23, 26, 49, 92, 103]. Low expression of DDX3X is correlated with poor prognosis in lung cancer, CRC and the non-smoking subpopulation of patients with HNSSC [49, 92, 103]. It is worth noting that the reduction in DDX3X expression is closely related to virus infection in lung cancer and HCC [23, 26]. In addition, in HCC, reduced DDX3X expression is more common in males than in females [23] In many cancers, DDX3X is predominantly present in the cytoplasm of cancer cells, whereas paired non-tumour tissue expresses little or no DDX3X. Nuclear localization of DDX3X has been detected in breast and colorectal cancer tissues and is correlated with other factors associated with poor prognosis [48, 114]. More importantly, patients with nuclear DDX3X expression have a worse prognosis than those without nuclear DDX3X [48].

### Cell cycle — evading growth suppressors

**Fig. 3:**
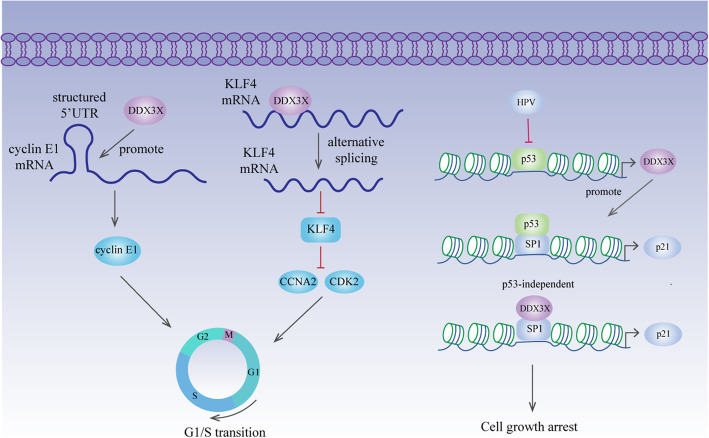
DDX3X and cell cycle. DDX3X facilitates cyclin E1 translation by resolving the secondary structure in its 5′UTR during translation initiation. Moreover, it suppresses KLF4 expression by manipulating KLF4 mRNA alternative splicing. Therefore, by promoting cyclin E1 translation and suppressing KLF4 expression, DDX3X promotes G1/S transition. In lung cancer, P53 promotes DDX3X transcription. DDX3X synergistically enhances p53-activated P21 transcription by increasing the binding affinity of SP1 on the P21 promoter. In liver cancer, DDX3X directly interacts with SP1 to promote P21 transcription in a P53-independent manner, thus leading to tumour cell growth arrest

The cell cycle is regulated by cyclins, CDKs (cyclin-dependent kinases) and CKIs (cyclin-dependent kinase inhibitors) [115]. Depletion of DDX3X induces G1 phase arrest in breast cancer, lung cancer, colorectal cancer, prostate cancer and medulloblastoma [79, 89, 97, 98, 104]. This phenomenon might result from a reduction in cyclin E1, which is upregulated by DDX3X at the translation level (Fig. 3) [33]. Additionally, DDX3X inhibits the expression of KLF4 by altering the alternative splicing of KLF4 mRNA, followed by upregulated CCNA2 and CDK2 expression [97] (Fig. 3). P21 is a classic CKI that causes cell growth arrest by interacting with cyclin/CDK complexes [116]. P53 transactivates the P21 promoter via increased SP1 binding affinity [117]. In lung cancer, DDX3X transcription is directly regulated by P53 [92]. More importantly, DDX3X synergistically enhances P53-activated P21 transcription by increasing the interaction between P53 and SP1 and promoting SP1 binding to the P21 promoter [92] (Fig. 3). In E6-positive lung tumours, downregulation of DDX3X expression by P53 inactivation promotes cell proliferation and colony formation via reduced SP1 binding activity on the P21 promoter [92]. However, P21 expression can also be induced in a P53-independent manner [118]. In liver cancer, the reduction in P21 is independent of P53 status, and DDX3X directly interacts with SP1 to promote P21 transcription, leading to tumour cell growth inhibition [20] (Fig. 3). Nevertheless, reduced P21 expression caused by DDX3X overexpression is also observed in breast cancer [24].

### Apoptosis — resisting cell death

**Fig. 4:**
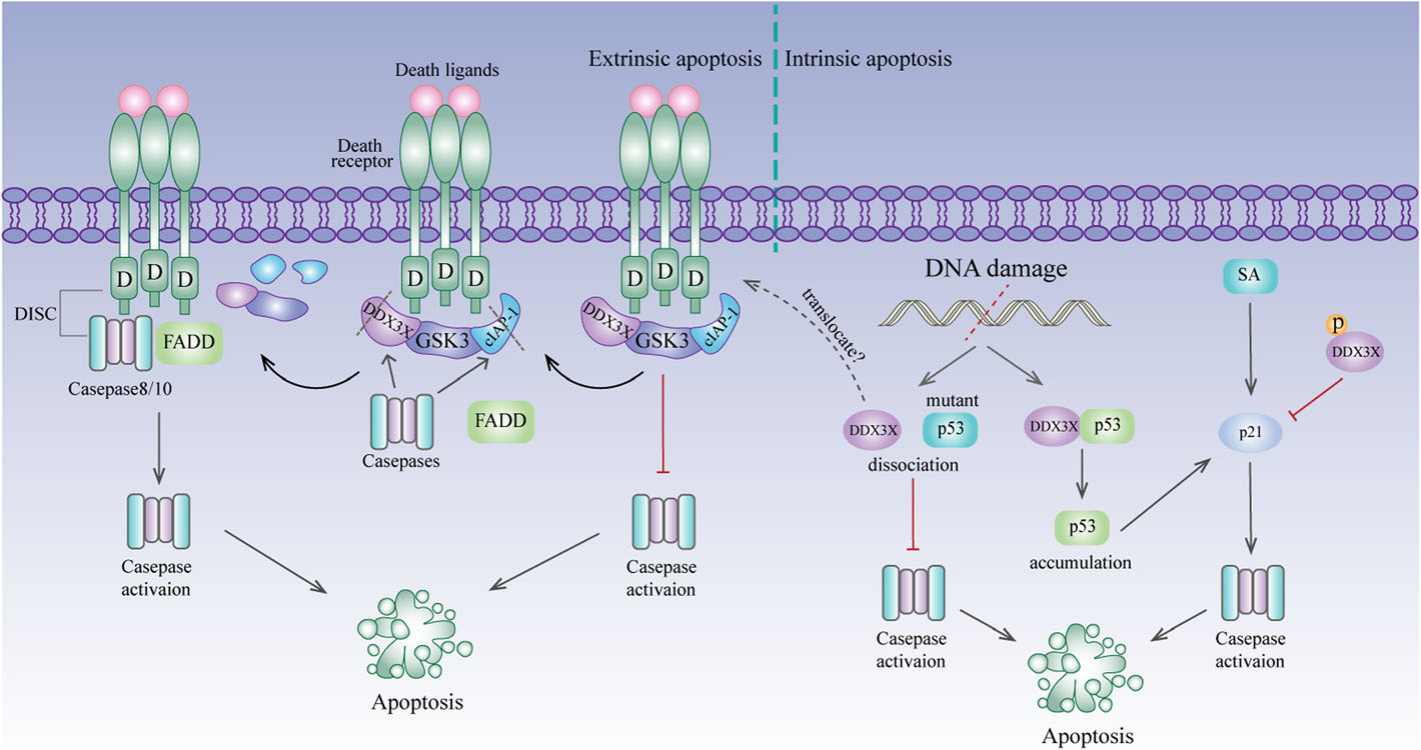
DDX3X and apoptosis. In the extrinsic apoptosis pathway, DDX3X binds with GSK3 and cIAP-1, forming an anti-apoptotic complex to cap major death receptors. After death ligands bind to their receptors, the anti-apoptotic complex is destroyed by inactivation of GSK3β and cleavage of DDX3X and cIAP-1 by caspases. Cleavage occurred in the N-terminus of DDX3X, and the truncated protein can still bind GSK3-β. In the intrinsic apoptosis pathway, DDX3X binds wild-type P53 and mutant P53 in tumours. When encountering DNA damage, DDX3X binds wild-type P53 and stabilizes its protein level, promoting P53/P21 axis-mediated apoptosis. However, DDX3X and mutant P53 are separated after DNA damage occurs, which impedes Caspase activation in P53-mutant tumours. This disassociation may contribute to the translocation of DDX3X to death receptors. However, in HeLa cells, phosphorylated DDX3X reverses sanguinarine (SA)-induced intrinsic apoptosis by strongly repressing P21 expression

Apoptosis is a process of programmed cell death that proceeds via the mitochondrial pathway (intrinsic) or the death receptor pathway (extrinsic) [119]. Extrinsic apoptosis is induced by the activation of death receptors. Stimulation of these receptors by death ligands results in the recruitment of FADD (Fas associated with death domain protein) and Caspase-8 (or Caspase-10) to form DISC (death-inducing signalling complex), thus promoting the activation of downstream Caspases [120]. DDX3X binds with TRAIL-R2 and is cleaved during TRAIL-mediated apoptosis [34]. In addition, DDX3X cooperates with GSK3 and cIAP-1 to form an anti-apoptotic complex that caps major death receptors before they can be stimulated [45]. Stimulated death receptors overcome the anti-apoptotic cap by inactivating GSK3β and cleaving DDX3X and cIAP-1 [45]. Cleavage occurs in the N-terminus of DDX3X, and the truncated protein can still bind GSK3-β [45]. However, the complex remains functional in cancer cells resistant to death receptor stimulation (Fig. 4). Collectively, an inability of the death receptors to disable DDX3X activity may contribute to resistance to death receptor-induced apoptosis in tumours, suggesting that targeting DDX3X might be a useful strategy for promoting death receptor-induced apoptosis.

P53 plays a vital role in DNA damage-induced intrinsic apoptosis [121, 122]. DDX3X binds with both wild-type P53 and mutant P53 in tumours. When DNA damage occurs, DDX3X can still bind wild-type P53 and stabilize its protein level, thus promoting P53-mediated apoptosis. However, the association of DDX3X and mutant P53 was reduced after DNA damage occurred, which impeded Caspase activation [112]. Disassociation may contribute to the translocation of DDX3X to death receptors, where it attenuates Caspase activation (Fig. 4). Alterations in P21, a target gene of P53, are also observed when DDX3X is manipulated [112], thus confirming that DDX3X functions in intrinsic apoptosis via the DDX3X/P53/P21 axis. However, another report showed that phosphorylated DDX3X reverses sanguinarine-induced intrinsic apoptosis by strongly repressing P21 expression [113] (Fig. 4). The different results from these two reports might be due to different drug models or cell types.

### Metastasis — activating invasion and metastasis

**Fig. 5:**
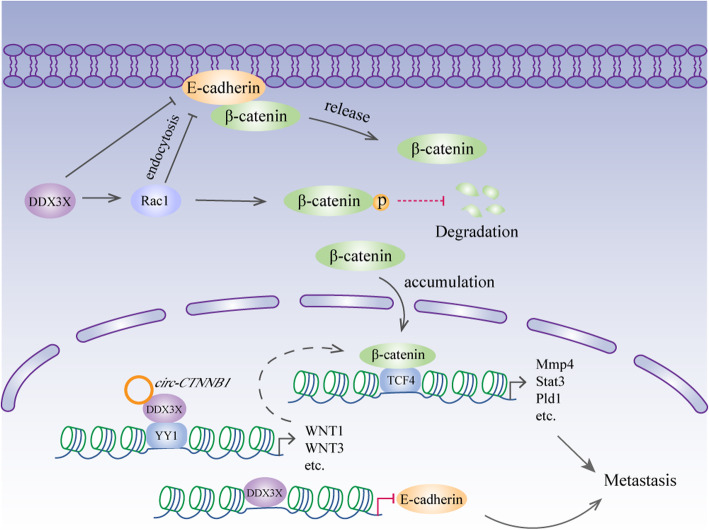
DDX3X and metastasis. Rac1 protects β-catenin from degradation by enhancing β-catenin phosphorylation on S675. DDX3X facilitates Rac1 translation to stabilize β-catenin. Additionally, DDX3X might modulate cell-cell adhesions by enhancing Rac1-dependent E-cadherin endocytosis. Moreover, DDX3X directly binds to the E-cadherin promoter to suppress its expression. The decrease in E-cadherin expression causes β-catenin to be released from the cell membrane. The accumulated β-catenin translocates to the nucleus and interacts with TCF4 to increase the expression of its downstream target genes. In the nucleus, DDX3X activates the transcription factor YY1 with the help of circRNA circ-CTNNB1, leading to subsequent activation of genes involved in WNT/β-catenin signalling

DDX3X and β-catenin are very closely related in metastasis. Chen et al. reported that DDX3X promotes cancer cell migration and invasion via the Rac1/β-catenin pathway [36]. Rac1 plays a pivotal role in cell-cell contacts and cell migration [123]. Importantly, it protects β-catenin from proteasome-dependent degradation by enhancing β-catenin phosphorylation on S675 [124]. By facilitating the translation of Rac1 mRNAs containing a structured 5′UTR, DDX3X stabilizes β-catenin, thus increasing the expression of its downstream transcriptional targets involved in tumour metastasis, including MMP14, Pld1 and Stat3 [36]. DDX3X also modulates cell-cell adhesion by downregulating E-cadherin [36], which might be achieved by enhancing Rac1-dependent E-cadherin endocytosis [125]. Therefore, DDX3X likely promotes metastasis through both the Rac1/E-cadherin and Rac1/β-catenin pathways (Fig. 5). Of note, E-cadherin is negatively regulated by the transcription factor snail [126]. DDX3X can induce Snail expression to suppress E-cadherin expression and drive metastasis [78]. In addition, the helicase directly binds to the E-cadherin promoter and represses its transcriptional activity [24]. E-cadherin, along with the majority of β-catenin, predominantly localizes to the cell membrane. Depletion of E-cadherin induced by DDX3X abolishes this interaction and releases β-catenin into the cytoplasm and nucleus [24]. Therefore, the motility and invasive properties induced by DDX3X are probably mediated by direct and indirect mechanisms (Fig. 5). In gastric cancer, DDX3X binds the transcription factor YY1 (yin yang 1) with the help of the circRNA *circ-CTNNB1*, which results in the transactivation of YY1 and the subsequent activation of genes involved in WNT/β-catenin signalling, thereby promoting tumour progression (Fig. 5) [51].

**Fig. 6:**
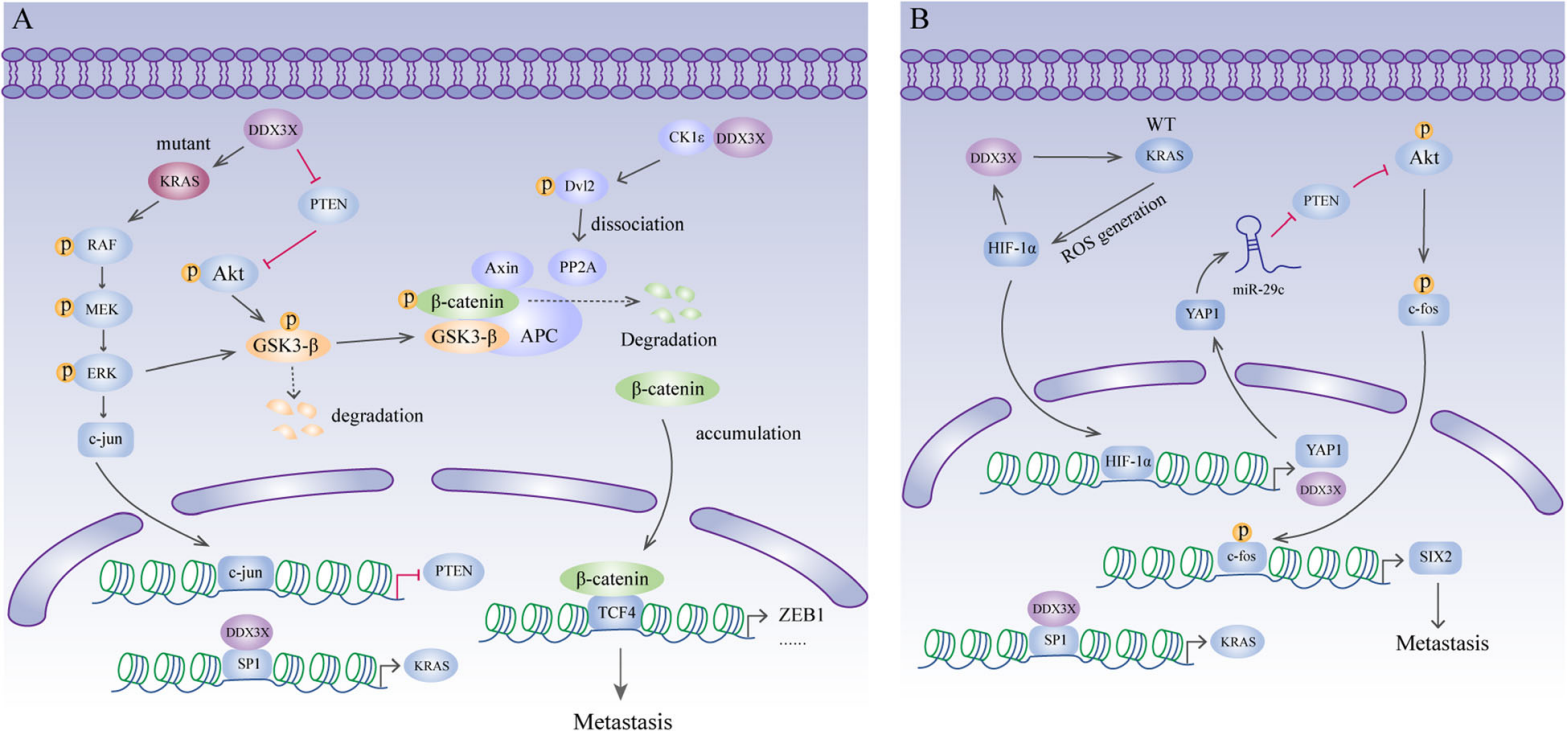
DDX3X and metastasis. **a** In colorectal cancer, DDX3X increases the expression of KRAS by promoting SP1 binding to the KRAS promoter. In CRC harbouring mutant KRAS, DDX3X activates the KRAS/ERK/PTEN/AKT cascade to stabilize β-catenin, which then enhances ZEB1 transcription to promote metastasis. In addition, DDX3X binds with and activates CK1ε, which then phosphorylates Dvl2. Phosphorylated Dvl2 causes dissociation of PP2A and the β-catenin degradation complex, therefore stabilizing β-catenin. The accumulated β-catenin translocates into the nucleus and interacts with TCF4 to increase the expression of its downstream target genes. **b** In CRC harbouring wild-type KRAS, DDX3X/KRAS/HIF1-α generates a cascade feedback loop. HIF-1α binds to the YAP1 promoter to promote YAP1 transcription. YAP1 then targets PTEN by elevating miR-29c expression to activate PI3K/AKT signalling. Phosphorylated AKT activates c-fos and eventually leads to increased levels of SIX2 transcription

KRAS is an oncogene that is mutated in more than 40% of human colorectal cancer cases [127]. In colorectal cancer, DDX3X increases the expression of KRAS by promoting SP1 binding to the KRAS promoter to facilitate tumour metastasis [25, 128]. However, the specific molecular mechanisms by which DDX3X promotes metastasis are different in colorectal cancers harbouring wild-type or mutant KRAS. In colorectal cancer harbouring mutant KRAS, DDX3X-induced expression of KRAS activates the RAF/MEK/ERK/c-Jun pathway to suppress the tumour suppressor gene PTEN, a negative regulator of the PI3K/AKT pathway [100]. GSK3-β plays a crucial role in β-catenin degradation via phosphorylation on the N-terminus of β-catenin [129]. However, the activity of GSK3-β can be suppressed by the MEK/ERK and PI3K/AKT signalling pathways [130, 131]. Therefore, DDX3X deactivates GSK3-β to stabilize β-catenin, which then enhances ZEB1 transcription for metastasis [25] (Fig. 6a). Additionally, in colorectal cancer harbouring mutant KRAS, DDX3X stabilizes β-catenin via the CK1ε/Dvl2 axis to promote invasiveness [101]. Similar to the abovementioned findings, DDX3X binds CK1ε and stimulates its activity. Activated CK1ε then phosphorylates Dvl2, which decreases the association of PP2A with the β-catenin degradation complex, therefore stabilizing β-catenin [101] (Fig. 6a). In colorectal cancer harbouring wild-type KRAS, DDX3X-induced KRAS elevated the level of ROS, which was followed by increased HIF1-α expression. HIF-1α in turn directly upregulates DDX3X expression at the transcriptional level, thus generating a cascade feedback loop [128]. Furthermore, DDX3X-induced HIF-1α directly binds to the YAP1 promoter to promote its transcription [128]. YAP1, a novel oncogene in the Hippo pathway, targets PTEN by elevating miR-29c expression to activate PI3K/AKT signalling [132]. The activated PI3K/AKT pathway upregulates the expression of and phosphorylates the transcription factor c-fos and eventually leads to the increased transcription level of SIX2, a gene that suppresses E-cadherin expression to promote metastasis in breast cancer [102]. The YAP1/SIX2 axis is responsible for DDX3X-induced cell invasiveness in colorectal cancer harbouring wild-type KRAS (Fig. 6b). In addition to promoting metastasis, the DDX3X-induced YAP1/SIX2 axis might be responsible for resistance to treatment with the anti-EGFR antibody cetuximab (CTX) in colorectal cancer harbouring wild-type KRAS via enhanced autophagy and anti-apoptotic mechanisms [128]. However, Su et al. reported that downregulation of DDX3X expression in colorectal cancer leads to upregulation of Snail expression, decreased E-cadherin expression and increased vimentin and N-cadherin expression. Furthermore, knocking down Snail significantly reduced the migration and invasion capacities of cells with DDX3X knockdown, indicating that DDX3X represses colorectal cancer cell metastasis by mediating the Snail/E-cadherin pathway [103]. These conflicting results in colorectal cancer are possibly due to the use of different cell lines. However, the actual role of DDX3X in colorectal cancer needs to be verified.

**Fig. 7:**
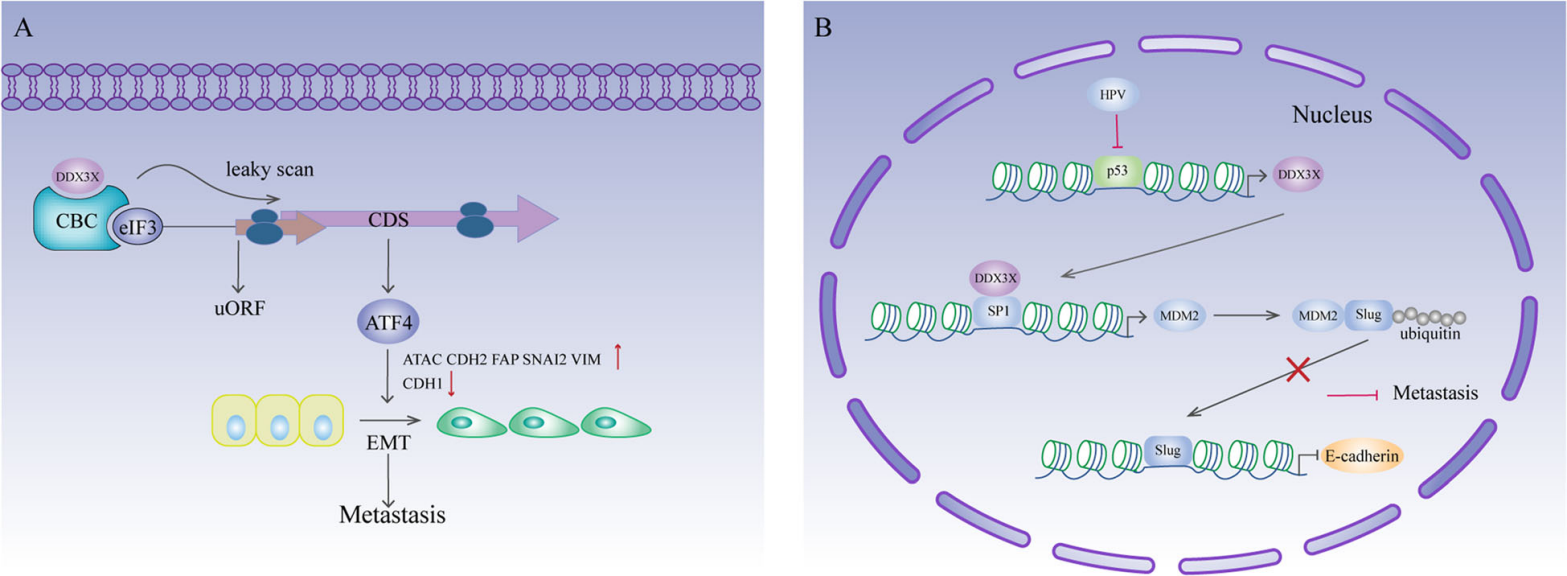
DDX3X and metastasis. **a** In HNSCC, DDX3X cooperates with the CBC-eIF3 complex to enhance the translation of ATF4 mRNA. Increased ATF4 expression results in upregulated expression of ACTA2, CDH2 (N-cadherin), FAP, SNAI2 (Slug), and VIM (vimentin) and downregulated expression of CHD1 (E-cadherin), thus triggering EMT. **b** DDX3X interacts with SP1 to promote MDM2 transcription. In E6-positive lung cancer, P53 inactivation leads to downregulation of DDX3X expression, which suppresses MDM2 expression. MDM2 promotes E-cadherin expression by mediating proteasomal degradation of Slug. Therefore, loss of DDX3X downregulates MDM2 expression, stabilizes Slug and suppresses E-cadherin expression, which eventually promotes tumour metastasis

In head and neck squamous cell carcinoma (HNSCC), DDX3X cooperates with the CBC-eIF3 complex to enhance some uORF-containing mRNAs [29]. ATF4 is a crucial gene for EMT [133–135] and is responsible for the effect of DDX3X overexpression on EMT-related gene expression, including upregulation of ACTA2, CDH2 (N-cadherin), FAP, SNAI2 (Slug), and VIM (vimentin) expression and downregulation of CHD1 (E-cadherin) expression. In addition, knockdown of CBC or eIF3 impairs cell invasiveness and decreases the expression of mesenchymal-related genes but increases the expression of E-cadherin [29]. Therefore, there is a possibility that DDX3X acts co-ordinately with the CBC-eIF3 complex to enhance the translation of mRNAs containing uORFs that together modulate the EMT program, hence promoting HNSCC metastasis (Fig. 7a). In lung cancer, loss of DDX3X via P53 inactivation suppresses MDM2 transcription by decreasing SP1 binding to the MDM2 promoter [26]. MDM2 promotes E-cadherin expression by mediating proteasomal degradation of Slug [136]. Loss of DDX3X stabilizes Slug expression by suppressing the MDM2-mediated ubiquitin proteasomal pathway and consequently suppresses E-cadherin expression, thus promoting cell invasion [26] (Fig. 7b). Melanoma is an aggressive malignancy of melanocytes characterized by rapid metastasis [137]. A mutational assessment of 864 melanoma tumours identified DDX3X mutations, most of which might eventually lead to DDX3X loss of expression. Intriguingly, loss of DDX3X expression directs a proliferative-to-metastatic phenotypic switch in melanoma cells [30].

MITF, which is strongly correlated with a less invasive and more proliferative expression signature in melanoma [138], was identified as a direct translational target of DDX3X [30]. Mechanistically, DDX3X promotes MITF mRNA translation via its internal ribosome entry site (IRES) within the 5′UTR.

### Gene instability and mutation

In recent years, mutations in DDX3X have been reported to be involved in chronic lymphocytic leukaemia (CLL), an incurable disease with variable clinical presentation and evolution [106–108, 139]. In 48 CLL cases, 10% (5/48) presented with DDX3X mutations, which were either nonsense mutations or frameshift indels that eventually led to truncated production. Moreover, DDX3X is preferentially mutated in males (4/5). Furthermore, in two of the five male cases, two independent truncating mutations were identified. Analysis of these two cases shows that these mutations showed trends increased and decreased activity at different time points [106], Additionally, inactivating DDX3X mutations are associated with unfavourable clinical markers and poor clinical outcomes [106]. A longitudinal analysis in a cohort of 8 cases of monoclonal B-cell lymphocytosis (MBL) showed that one case possessed a mutation in SF3B1 and two independent mutations in DDX3X. Longitudinal analysis of this patient demonstrates that at the first time point, DDX3X mutation I415V was present in nearly 50% of the allelic fraction, while the mutation D164G was present in only 10%. However, at the second time point, this trend was reversed [140]. The variation in DDX3X mutations in CLL and MBL suggests the presence of DDX3X mutations in different subclones with alternating dominance between the time points. MLLT10 is a moderately common MLL fusion partner that predominantly occurs in acute monoblastic leukaemia (AML) [141]. DDX3X is one of the partners of MLLT10 in adult and paediatric T-cell acute lymphoblastic leukaemia (T-ALL) [31, 109]. Upon investigating 99 patients with adult T-ALL, researchers found that approximately 10% (10/99) of the patients had MLLT10 translocations. Among them, 3 cases possessed the DDX3X-MLLT10 fusion. Another biological sample from a 4th patient was obtained from a different cohort of 20 adult T-ALL patients. All 4 cases were confirmed to have in-frame DDX3X-MLLT10 transcripts with different breakpoints. At the N-terminus, DDX3X contains a nuclear export signal (NES) domain. Three patients retained the entire eIF4E interacting motif, and 1 retained only half of this motif. The MLLT10 leukaemogenic OM-LZ domain, which induces acute myeloid leukaemia in mouse models, was maintained at the C-terminus in all the fusions. In addition, all 4 cases were males, indicating that the complete absence of a normally functional DDX3X protein might contribute to leukaemogenesis [31]. In natural killer/T-cell lymphoma (NKTCL), whole-exome sequencing in 25 patients and subsequent target sequencing in 80 patients show that recurrent mutations are most frequently located in DDX3X (20.0%, 21/105), followed by P53, STAT3, etc. Most of the mutations in DDX3X affect two highly conserved RacA-like domains. Half of the mutations eventually lead to truncation or loss of the protein, while the other half lead to altered protein function. Indeed, DDX3X with the mutations A404P and E348K exhibits decreased RNA-unwinding activity, an impaired ability to suppress cell cycle progression and abnormally activated NF-κB and MAPK pathways at the transcriptional level. In addition, DDX3X mutations are correlated with advanced disease stage and poor clinical outcome [42]. It is worth noting that DDX3X and P53 are the two genes most commonly mutated in NKTCL, but they seldom overlap with each other, implying that they are involved in very closely related biological processes in NKTCL. Mutations in DDX3X have also been discovered in aggressive natural killer-cell leukaemia (ANKL), a rare mature NK-cell tumour [110]; Burkitt lymphoma (BL) [161]; and Burkitt-like lymphoma with 11q aberration (BLL-11q), a category similar to Burkitt lymphoma but lacking the *MYC* rearrangement and containing 11q arm distortion [111].

Medulloblastoma (MB) arises in the cerebellum or medulla/brain stem [142] and is the most common malignant childhood brain tumour [143]. In the last few years, gene expression profiling of moderate-to-large cohorts of patients with this disease identified 4 distinct molecular subgroups: WNT, presenting wnt pathway activation; SHH, displaying hedgehog pathway activation; and groups 3 and 4, which are less well characterized on the molecular level [32]. The results from a wave of medulloblastoma genome-sequencing studies revealed that DDX3X is the second most frequently mutated gene in medulloblastoma (8%, 25/300), followed by CTNNB1 (β-catenin). Half of WNT medulloblastoma patients from three cohorts harboured DDX3X variants (50%, 16/32), while the percentage of patients with SHH medulloblastoma was 11% (7/66) [80–82]. Another genome sequencing analysis of SHH medulloblastoma showed that DDX3X is mutated in 54% of adult SHH medulloblastomas (27/50) and 7.2% of paediatric medulloblastomas (6/83) [144]. In contrast to mutations found in blood cancer that contain premature stop codons, frameshifts, or splice variants, nearly all mutations in medulloblastoma were nonsynonymous single nucleotide variants (SNVs), which were likely to cause alteration of protein function rather than loss of function [80–82]. Indeed, neither wild-type DDX3X nor mutant DDX3X enhanced the ability of β-catenin to transactivate TCF/LEF in medulloblastoma. However, the majority of DDX3X mutations enhance cell proliferation by potentiating the transactivation capacity of mutant β-catenin [81]. Moreover, mutations in DDX3X are crucial for the proliferation and/or maintenance of the LRLP lineage, which is believed to be the cell-of-origin of WNT medulloblastoma [80]. The variants of DDX3X appeared to cluster in either of the two helicase domains, which are important for catalytic function [80–82]. Consistently, further functional studies on mutations in medulloblastoma revealed that DDX3X mutants G302V and G325E have severely defective RNA-stimulated ATPase activity and cannot complement the growth defect in a Ded1p (yeast homologue of DDX3X) temperature-sensitive strain of fission yeast [41]. Moreover, mutations in DDX3X were confirmed to drive stress granule assembly and impair global translation [145]. Wild-type DDX3X interacts extensively with RNA and ribosomal machinery to help remodel the translation landscape in response to stress, while DDX3X with the mutation R534H adapts this response to selectively preserve translation involved in chromatin organization [146]. In melanoma, DDX3X was mutated in 5.8% of the 864 tumours. These mutants included 35% truncating mutations and 65% missense mutations, which might eventually lead to the loss of DDX3X expression. Importantly, 82% of all DDX3X mutations, including all truncating mutations, were detected in male patients, implying that DDX3X might play an important role in the progression of melanoma in males [30]. In addition to the aforementioned malignancies, DDX3X mutations were also found in progressive/higher grade meningiomas and mesotheliomas [84, 93, 94]. DDX3X CNVs (copy number variants) were also found in patients with oral squamous cell carcinoma (OSCC) [147].

### Deregulating cellular metabolism

Mitochondrial localization of DDX3X has been discovered in breast cancer and colorectal cancer cells [50, 99]. Targeting DDX3X inhibits mitochondrial translation, followed by reduced oxidative phosphorylation (OXPHOS) and increased ROS (reactive oxygen species) production, which ultimately triggers apoptosis and causes cell death [50, 99]. Cellular stressors, such as ionizing radiation, can also increase ROS expression. In addition, ionizing radiation leads to an increased demand for ATP in cancer cells, which needs to be addressed by a large OXPHOS reserve capacity [148, 149]. The combination of ionizing radiation and DDX3X inhibition causes cancer cells to undergo metabolic catastrophe [50], which is a promising anti-tumour therapeutic strategy. In breast cancer, DDX3X expression is induced by HIF-1 under hypoxic conditions [96]. Its expression is also correlated with other hypoxia-responsive genes [95]. This evidence indicates that DDX3X plays a role in hypoxia, but the specific function of DDX3X in these conditions needs to be further explored.

### Stemness and immunogenicity — tumour microenvironment

**Fig. 8:**
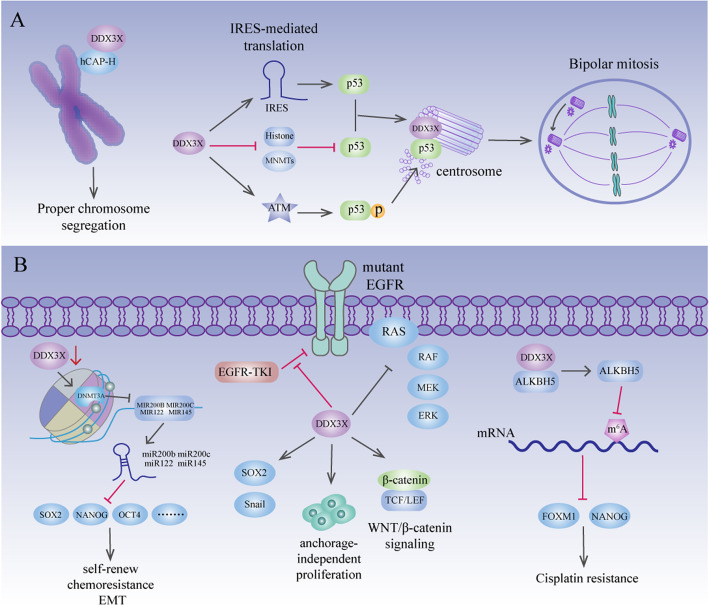
DDX3X in mitosis and stemness. **a** During prophase/prometaphase of mitosis, DDX3X translocates in close proximity to the condensing chromosomes and interacts with hCAP-H to promote chromosome segregation. In colon cancer and osteosarcoma, DDX3X upregulates P53 expression by promoting IRES-mediated translation of P53, thus preventing DNMTs from hypermethylating the P53 promoter and repressing the binding of repressive histone markers to the P53 promoter. In addition, DDX3X activates ATM kinase to phosphorylate P53, which leads to the localization of P53 to centrosomes. At the centrosome, DDX3X interacts with P53, leading to inactivation and coalescence of excess centrosomes. **b** In liver cancer, DDX3X upregulates the expression of miR-200b, miR-200c, miR-122 and miR-145 by reducing DNMT3A binding and hypermethylation of their promoter regions. This subset of miRNAs suppresses the expression of signature stemness genes, including SOX2, NANOG, and OCT4, which are responsible for self-renewal, chemoresistance and EMT. In lung adenocarcinoma cells harbouring an EGFR mutation, DDX3X overexpression induces increased Sox2 and Snail expression, anchorage-independent proliferation, resistance to EGFR-TKIs and promotion of Wnt/β-catenin signalling. In cisplatin-resistant OSCC cells, DDX3X interacts with ALKBH5 and increases its expression. ALKBH5 then upregulates FOXM1 and NANOG expression by demethylating their methylated mRNAs, which eventually leads to cisplatin resistance

Cancer stem cells (CSCs) are a subset of cells within a tumour that are responsible for the long-term maintenance of tumour growth in several cancers [150]. CSCs are characterized by self-renewal, chemoresistance, EMT, motility and CSC expansion, which result in tumour initiation and anti-cancer therapy resistance [151]. Human small cell lung carcinoma, colorectal cancer, and breast cancer cells with CSC markers express a high level of DDX3X, yet normal human tissues only faintly express DDX3X [88]. In liver cancer, well-differentiated cell lines showed higher expression of DDX3X than did poorly differentiated cell lines. Moreover, DDX3X represses the expression of signature stemness genes, including NANOG, OCT4, c-MYC, SOX2, KLF4, BMI1 and CK19, to prevent the generation of CSCs in liver cancer [66]. Mechanistically, DDX3X represses the expression of stemness genes via upregulation of the expression of a subset of tumour-suppressive miRNAs, including miR-200b, miR-200c, miR-122 and miR-145, by reducing DNMT3A (DNA methyltransferase 3A) binding and hypermethylation on their promoter regions [66] (Fig. 8a). Conversely, in lung adenocarcinoma cells harbouring an EGFR mutation, DDX3X overexpression induces a CSC-like phenotype (increased Sox2 and Snail expression and elevated anchorage-independent proliferation) and resistance to EGFR-tyrosine kinase inhibitors (EGFR-TKIs) [91]. Overexpression of DDX3X reduces EGFR signalling but facilitates Wnt/β-catenin signalling [91], which is consistent with the fact that stem cells can utilize Wnt/β-catenin signalling pathways to replace receptor-type tyrosine kinase signalling [152]. In fact, control cells, which are nonadherent, lack EGFR signalling and resist EGFR-TKIs, express high levels of DDX3X [91], providing further proof that DDX3X plays a role in inducing a stem cell-like state (Fig. 8a). Consistent with the findings in lung cancer, DDX3X is also upregulated in cisplatin-resistant oral squamous cell carcinoma (OSCC) cells compared to cisplatin-sensitive OSCC cells [86]. Targeting DDX3X impairs the CSC population in cisplatin-resistant cells via decreased expression of FOXM1 and NANOG [86], which are important for self-renewal properties and drug resistance in cancers that are upregulated by the m^6^A demethylase ALKBH5 [43, 153–156]. In cisplatin-resistant OSCC cells, DDX3X interacts with ALKBH5 and increases its expression to upregulate FOXM1 and NANOG expression [86] (Fig. 8a). DDX3X is a major immunogenic protein in CD133^+^ melanoma cells. Inoculation with DDX3X-primed specific T cells exhibits defensive and beneficial antitumour immunity, curing established skin melanoma. DDX3X-primed CD4^+^ T cells produce tumour-specific IFNγ and IL-17 from CD133^+^ cells, suggesting that DDX3X possesses immunogenic MHC class II-restricted epitopes [88]. Therefore, anti-DDX3X immunotherapy is a promising treatment to eradicate CSCs to cure cancer.

### Mitosis

During mitosis, each daughter cell inherits one copy of every chromosome. The accuracy of this process is achieved by chromosome segregation, as mediated by condensin I and II complexes [157]. Defects in chromosome segregation cause aneuploidy and then cell death or cancer [158]. DDX3X regulates chromosome segregation by interacting with hCAP-H in HeLa cells [35]. hCAP-H is a subunit of condensin I that has been shown to promote proper chromosome segregation in HeLa cells [35]. During interphase, DDX3X localizes in cytoplasmic foci. In prophase/prometaphase, DDX3X translocates within close proximity to the condensing chromosomes and interacts with hCAP-H to promote chromosome segregation. Knockdown of DDX3X abolishes the robust localization of hCAP-H to mitotic chromosomes, leading to an increased incidence of lagging chromosomes [35] (Fig. 8b). In colorectal cancer and osteosarcoma, DDX3X prevents multipolar mitosis through inactivation and coalescence of excess centrosomes [4]. Interestingly, the localization of DDX3X to centrosomes is dependent on P53 expression Moreover, DDX3X promotes IRES-mediated translation of P53, and DDX3X knockdown activates DNMTs to hypermethylate the P53 promoter and promotes the binding of repressive histone markers to the P53 promoter. DDX3X also promotes P53 Ser15 phosphorylation by activating ATM kinase, which eventually leads to the localization of P53 centrosomes [4]. Therefore, by regulating P53 expression and colocalizing with P53, DDX3X ensures proper mitotic progression and genome stability [4], implying its tumour-suppressive function (Fig. 8b).

### Tumorigenesis

Overexpression of DDX3X in the liver cancer cell line Tong leads to moderate colony formation in soft agar, whereas unadulterated Tong cells per se do not have this ability [90]. BPDE (benzopyrene diol epoxide), a major cancer-causing compound, induces consistent activation of DDX3X in the immortalized human breast cell line MCF10A. Overexpression of DDX3X in MCF10A cells leads to colony formation in soft agar assays, induction of EMT and enhanced cell motility and invasive properties [24]. Nonetheless, hepatocyte-specific DDX3X ablation promotes the development of hepatocellular tumours in aged female mice, whereas loss of DDX3X causes profound ductular reactions and apoptosis, followed by compensatory proliferation in young female mice. In addition, DNA single-strand break and double-strand break signalling are induced in young female mice with ablated DDX3X expression, indicating that replicative stress occurs. Furthermore, DDX3X is found to bind to the promoter regions of DDB2 and XPA, two DNA repair factors, via transcription factor SP1 to maintain genome stability [12]. Therefore, loss of DDX3X led to accumulated DNA damage and replication stress and eventually to spontaneous liver tumours and accelerated DEN-induced (diethylnitrosamine-induced) liver tumorigenesis [12].

### Anti-cancer therapy

RK-33 is a synthetic DDX3X inhibitor that specifically binds to the ATP-binding cleft of DDX3X and causes decreased unwinding activity. In lung cancer, RK-33 inhibits cell growth and sensitizes lung cancer cells to radiotherapy [89]. The combination of RK33 and radiation can effectively promote tumour regression [89]. Mechanistically, RK-33 inhibits Wnt/β-catenin pathway activity and impairs the response to radiation-induced DNA double-strand breaks (DSBs) [89]. Along with its effects on lung cancer, RK33, especially in combination with radiation, has been reported to exert inhibitory effects on many other cancers, including breast cancer, prostate cancer, colorectal cancer, medulloblastoma and Ewing sarcoma [50, 79, 98, 104, 105]. In addition to RK-33, other compounds, such as doxorubicin and ketorolac salt, have been confirmed to inhibit DDX3X and are ideal drug candidates to treat DDX3X-associated oral cancer [86, 87, 159]. Avenanthramide A (AVNs), an extract of oat bran, can directly bind to the Arg287 and Arg294 residues in DDX3X and repress protein expression. AVNs targeting DDX3X lead to mitochondrial swelling and increase ROS production, eventually leading to cell death in colorectal cancer [99].

## Conclusion

DDX3X possesses numerous functions in the processes of RNA metabolism, viral infection and cancer biology. Its roles in tumours are diverse and cancer-type dependent. There are many factors that influence the expression or functions of DDX3X, including viral infection, sex and cellular localization [12, 20, 23, 26, 49]. The expression of DDX3X is correlated with viral infection. In liver cancer, the reduction in DDX3X is positively linked with hepatitis virus infection, especially HBV [23]. In lung cancer, HPV-induced P53 inactivation causes loss of DDX3X expression [26]. Since DDX3X can directly or indirectly affects virus replication [160], it might participate in the progression of viral infection to oncogenesis. DDX3X is an X-linked gene that escapes X-inactivation in females [5]. This unique property of DDX3X might explain why cancers caused by a reduction or loss in DDX3X expression mostly occur in male patients [30, 31]. However, liver tumours induced by hepatocyte-specific DDX3X ablation mostly occurred in female mice [12], indicating the possibility that DDX3Y in male mice acts as a functional substitute for the loss of DDX3X in some contexts. DDX3X can shuttle between cytoplasm and nuclei. The subcellular localization of DDX3X seems to determine the fate of the tumour as well [16, 39, 48]. Most studies have indicated high cytoplasmic DDX3X expression in tumour tissues, and some of them indicate that this predicts poor prognosis [28, 29, 85, 89]. High expression of nuclear DDX3X was also present in colorectal cancer and breast cancer [48]. Nevertheless, nuclear DDX3X might also function as a sentinel to protect squamous cells from tumorigenesis [20, 49]. However, the concrete relationship between DDX3X and these factors needs to be further verified.

Numerous studies have uncovered the relationship between DDX3X and other functional proteins in cancer biology. P53 is a well-studied, powerful tumour suppressor. In lung cancer, P53 is located upstream of DDX3X and regulates DDX3X by directly activating the DDX3X promoter [92]. Moreover, these proteins work together to regulate the cell cycle, activation of intrinsic apoptosis and genome stability [4, 33, 92]. DDX3X also has a strong connection with oncogenic β-catenin. In normal mammalian cells, DDX3X activates β-catenin as a subunit of CK1ε in a wnt-dependent manner [105]. However, in multiple cancers, DDX3X activates β-catenin through a number of pathways to promote tumour progression [25, 36, 101]. Additionally, mutations in DDX3X frequently appear in WNT medulloblastoma, which is characterized by alterations of the WNT signalling pathway [80–82].

### Future research

The study of DDX3X in cancer involves in proliferation, metastasis, genome mutation and so on. Regrettably, the research of it in angiogenesis, immune destruction, tumour-promoting inflammation and cellular energetics are still in the initial stage. Although the functions of DDX3X have been gradually revealed, controversy persists as to whether regarding its identity as a tumour suppressor or oncogene has been ongoing. Thorough research is needed to settle this question. However, studies on DDX3X inhibitors and the achievements made so far have presented a new potential strategy for the treatment of cancer. It may be wise to advance the research of DDX3X targeted drug in cancer treatment.

## Data Availability

The datasets used and/or analysed during the current study are available from the corresponding author upon reasonable request.

## Abbreviations

ACTA2: Actin alpha 2
ALKBH5: RNA demethylase ALKBH5
ALS: Amyotrophic lateral sclerosis
ATF4: Cyclic AMP-dependent transcription factor ATF-4
ATIS: Alternative translation initiation site
ATP: Adenosine triphosphate
BL: Burkitt lymphoma
BMI1: Polycomb complex protein BMI-1
BPDE: Benzo pyrene diol epoxide
CBC: Cap-binding protein complex
CCNA2: Cyclin-A2
CDH2: Cadherin 2
CDK2: Cyclin-dependent kinase 2
CDK: Cyclin dependent kinase
c-fos: Proto-oncogene c-Fos
CHD1: Cadherin 1
cIAP-1: cellular inhibitor of apoptosis protein-1
CK19: Cytokeratin-19
CKI: Cyclin dependent kinase inhibitor
CK1ε: Casein kinase I isoform epsilon
CLL: Chronic lymphocytic leukaemia
CNVs: Copy number variants
CRC: Colorectal cancer
CRM-1: Chromosome region maintenance 1 protein homologue
CSCs: Cancer stem cells
cyclin E1: G1/S-specific cyclin-E1
CTX: Cetuximab
DDB2: DNA damage-binding protein 2
DDX3X (DBX, DDX3): ATP-dependent RNA helicase DDX3X
DDX3Y (DBY): ATP-dependent RNA helicase DDX3X
DISC: Death-inducing signalling complex
DEN: Diethylnitrosamine
DNMT3A: DNA methyltransferase 3A
DSB: Double-strand break
Dvl2: Segment polarity protein dishevelled homologue DVL-2
E-cadherin: Cadherin-1
eIF3: Eukaryotic translation initiation factor 3
eIF4A: Eukaryotic initiation factor 4A-I
eIF4E: Eukaryotic translation initiation factor 4E
eIF4G: Eukaryotic translation initiation factor 4 gamma 1
EJC: Exon junction complex
EMT: Epithelial-mesenchymal-like transformation
FADD: Fas associated with death domain protein
FAP: Fibroblast activation protein alpha
FOXM1: Forkhead box protein M1
FTD: Frontotemporal dementia
GSK3: Glycogen synthase kinase-3
hCAP-H: Condensin complex subunit 2
HCC: Hepatocellular carcinoma
HIF1-α: Hypoxia-inducible factor 1-alpha
HNSSC: Head and neck squamous cell
HRE: Hypoxia response element
IFN-β: Interferon beta
IFNγ: Interferon gamma
IL12: Interleukin-12
IRES: Internal ribosomal entry site
JUND: Transcription factor jun-D
KLF4: Krueppel-like factor 4
KRAS: GTPase KRas
MB: Medulloblastoma
MBL: Monoclonal B-cell lymphocytosis
MDM2: E3 ubiquitin-protein ligase Mdm2
MITF: Microphthalmia-associated transcription factor
MLLT10: Protein AF-10
Mmp14: Matrix metalloproteinase-14
mRNPs: ribonucleoproteins
MYC: Myc proto-oncogene protein
NANOG: Homeobox protein NANOG
NES: Nuclear export signal
NF-κB: Nuclear factor kappa-B
NKTCL: Natural killer/T-cell lymphoma
NLRP3: NACHT, LRR and PYD domains-containing protein 3
NMD: Nonsense-mediated decay
OCT4: Octamer-binding protein 4
OS: Overall survival
OSCC: Squamous cell carcinoma
OXPHOS: Oxidative phosphorylation
P21: Cyclin-dependent kinase inhibitor 1A
PDAC: pancreatic ductal adenocarcinoma
Pld1: Phospholipase D1
PTEN: Phosphatidylinositol 3,4,5-trisphosphate 3-phosphatase and dual-specificity protein phosphatase PTEN
PIC: Pre-initiation complex (PIC)
Rac1: Ras-related C3 botulinum toxin substrate 1
RAF: RAF proto-oncogene serine/threonine-protein kinase
RAN: Repeat-associated non-AUG
RFS: Relapse free survival
RNP: RNA binding protein
ROS: Proto-oncogene tyrosine-protein kinase ROS
RS-like: Arginine−/serine-like
SF3B1: Splicing factor 3B subunit 1
SGs: Stress granules
SIX2: Homeobox protein SIX2
Snail: Zinc finger protein SNAI1
SNAIL2: Snail family transcriptional repressor 2
SP1: Transcription factor Sp1
Stat3: Signal transducer and activator of transcription 3
SNVs: Single nucleotide variants
TAP: Nuclear RNA export factor 1
TBK1: TANK-binding kinase 1
TCF4: Transcription factor 4
T-ALL: T-cell acute lymphoblastic leukaemia
TRAIL: Tumour necrosis factor–related apoptosis-inducing ligand
uORFs: upstream open reading frames
VIM: Vimentin
XPA: DNA repair protein complementing XP-A cells
YAP1: Transcriptional coactivator YAP1
YY1: Transcriptional repressor protein YY1
ZEB1: Zinc finger E-box-binding homeobox 1
